# Effectiveness of Continuous and Sequential Chelation and Different Agitation Techniques on Smear Layer Removal and Microhardness of Root Canal Dentin (An In Vitro Study)

**DOI:** 10.3390/dj13050221

**Published:** 2025-05-20

**Authors:** Asmaa Aamir Kamil, Ahmed Hamid Ali, Federico Foschi, Francesco Mannocci

**Affiliations:** 1 Aesthetic and Restorative Dentistry Department, College of Dentistry, University of Baghdad, Baghdad 10071, Iraq; asmaa1994ak@gmail.com; 2 Unit of Endodontology, Department of Restorative Dentistry, UCL Eastman Dental Institute, University College London, London WC1E 6BT, UK; f.foschi@ucl.ac.uk; 3 Department of Endodontics, Centre of Oral Clinical & Translational Sciences, Faculty of Dentistry, Oral & Craniofacial Sciences, Guy’s Dental Hospital, King’s College London, London WC2R 2LS, UK; francesco.mannocci@kcl.ac.uk

**Keywords:** agitation, dual-rinse HEDP, EDTA, microhardness, smear layer

## Abstract

**Background/Objectives**: This study aimed to assess and compare the elimination of the smear layer and microhardness of dentin in root canals after sequential versus continuous chelation using different agitation techniques. **Methods**: Sixty-four palatal roots of upper first molars were instrumented to size X3 (Protaper Next files). According to the irrigant solution, samples were assigned to two groups (N = 32/group), 3% NaOCl irrigation followed by 17% EDTA (sequential chelation (SC)), or dual-rinse (3% NaOCl/9% HEDP) irrigation (continuous chelation (CC)). Each group has been divided into four subgroups (n = 8/subgroup), based on agitation techniques used: conventional needle (CN) (control group), EndoActivator (EA), ultrasonic agitation (UAI), and Er.Cr.YSGG 2780 nm (laser). SEM images assessed the smear layer, and Vicker microhardness (VHN) was performed at 50 and 100 µm depths. Data were analyzed using: Kruskal–Wallis, Wilcoxon, and the Mann–Whitney U test. Statistical significance was set at *p* < 0.05. **Results**: In the UAI and laser agitation, CC significantly reduced the smear layer presence compared to SC in the apical and coronal thirds, respectively (*p* < 0.05), and no significant differences were observed in the CN and EA groups between SC and CC (*p* > 0.05). There were significantly higher VHNs of dentine in CC groups than in SC groups in all sections and depths, except in the apical of the CN group at 50 µm and the coronal section of EA and UAI groups at 100 µm. **Conclusions**: CC was comparable to SC in smear layer removal. CC had a less detrimental effect on dentin compared with SC.

## 1. Introduction

The main objective of root canal therapy is to treat or prevent apical periodontitis, which is an immunological response to microbial infection of the root canal system [1] by removing or inhibiting the growth of microorganisms via cleaning, shaping, and tri-dimensional obturation [2]. On mechanically prepared dentine surfaces, an amorphous layer of organic and inorganic detritus known as the “smear layer” is created [3]. Research has shown that removing the smear layer enhances the fluid-tight seal of filled root canals, and bacteria eradication facilitates the penetration of antimicrobials, sealants, and intracanal medications into dentinal tubules [4]. Sodium hypochlorite (NaOCl) and ethylene diamine tetraacetic acid (EDTA) solutions are usually utilized among the many root canal irrigants for the removal of organic and inorganic components of the smear layer, respectively [5]. NaOCl and EDTA are used alternately or subsequently. Hence, this approach necessitates two distinct solutions called “sequential chelation” [6]. However, several serious drawbacks persist, such as the rapid removal of active chlorine upon NaOCl interaction with EDTA [7]. Research has indicated that the biomechanical characteristics of root dentin can be negatively impacted by prolonged usage of strong chelators like EDTA, as demonstrated by a decrease in microhardness and flexural strength [8]. While reports of irrigant interaction exist, applying this “sequential chelation” technique prevents irrigants from being mixed simultaneously, which might exacerbate their interaction [9].

Etidronic acid, also known as “1-Hydroxyethylidene-1, 1-Bisphosphonate” HEBP, or HEDP, is a soft biocompatible chelator used in direct conjunction with NaOCL to create a comprehensive solution that provides deproteinization, disinfection, and chelation functionalities [10]. A concept of continuous chelation uses the single solution of a weak chelator with sodium hypochlorite during the root canal instrumentation process without lowering NaOCL’s antibacterial and proteolytic activity [11]. The only available chelator presently offered as an approved commercial product, dual-rinse HEDP, is acceptable for clinical usage in endodontic treatment [12]. The dual-rinse HEDP product (Medcem, GmbH, Weinfelden, Switzerland) had been approved clinically based on this chemistry. The product comes in a 0.9 g etidronate capsule that needs to be combined with a 10 mL NaOCL solution [13].

The chemo-mechanical preparation markedly lowers bacterial load by directly targeting the root canal’s wall and facilitating the diffusion of antibacterial solutions into the dentinal tubules [14]. To enhance canal irrigation effectiveness and reduce the presence of refractory bacteria, various irrigant agitation methodologies have been devised [15]. Hand files, gutta–percha cones, plastic tools, sonic and ultrasonic devices, apical negative pressure irrigant administration, and photon-initiated photoacoustic streaming are some of these methods [16]. The sonically generated vibration of a polymer tip in a well-formed and fluid-filled channel causes a hydrodynamic phenomenon and the generation of intracanal waves [17]. In passive ultrasonic irrigation (PUI), energy is conveyed from an oscillating smooth wire through ultrasonic waves that generate acoustic streaming and may create cavitation in the irrigant, facilitating dynamic and comprehensive movement of the solution within the canal system [18]. The dentinal walls of the root canal can be effectively cleaned of smear layers and debris using Er,Cr-YSGG lasers operating at 2780 nm and Er: YAG lasers operating at 2940 nm. Both can cut hard tissue and allow for direct ablation of the root canal’s wall [19]. The development of numerous clinical protocols for liquid agitation using lasers has resulted from investigating the application of laser energy to improve the irrigant flow rate and create physical pressures against canal walls that can remove the smear layer, even in canals that have been minimally prepared [20]. Although applying a chelator and NaOCl together may have therapeutic advantages, the root dentin matrix may potentially be adversely affected. Prior studies have demonstrated that the use of NaOCl followed by EDTA or HEDP decreases the microhardness, flexural strength, fracture resistance, and erosion of the root canal dentin [9].

There is little information in the literature about the impact of dual-rinse HEDP irrigation solution with agitation on smear layer removal and the microhardness of dentin. Some studies have been conducted to assess the effects of different agitation methods using HEDP irrigant on the elimination of the smear layer [21]. The present study was conducted to assess and compare the efficacy of smear layer removal and the microhardness of prepared root canal dentin using sequential chelation SC (3% NaOCl followed by 17% EDTA) versus continuous chelation CC (3% NaOCl/9% HEDP) using a conventional needle, endoactivator, and ultrasonic and laser-agitation techniques. The null hypothesis proposed no difference in the smear layer removal and microhardness of the root canal between chelators with different agitation techniques used.

## 2. Materials and Methods

### 2.1. Sample Selection

The study involved 64 extracted human maxillary first molars with age range (18-to-35 years old) of patients to minimize variations in dentin hardness that were extracted. Power calculation of the study sample was based on 95% power with an alpha error of 5% and an effect size of 1.16; eight samples were required for each group [22]. The research ethics committee of the College of Dentistry, University of Baghdad, reviewed and approved the research project (project number 851523, ref. number 851 on 23 November 2023). Every tooth had a straight palatal root, while the roots were free from cracks, external root resorption, or fractures.

### 2.2. Sample Preparation

Calculus was removed using an ultrasonic scaler. The teeth were then submerged for 48 h in a 1% thymol solution (Sigma–Aldrich, Steinheim, Germany) and kept in deionized water until the designated procedure was initiated. After sectioning the palatal roots to a length of 12 mm, a size 10 K-file (Dentsply Maillefer, Ballaigues, Switzerland) was inserted into each root canal until it became visible at the apex. The working length was established by deducting 1 mm from the measured value. Size 10 of K-file (Dentsply Maillefer, Ballaigues, Switzerland) was used to achieve root canal accessibility.

The Endo Motor (E-CONNECT, 18th, Changzhou Sifary Medical Technology Co., Ltd., Changzhou, China) with a rotation speed of 300 RPM and 2 Ncm torque, with Protaper Next^®^ rotary files (Dentsply Maillefer, Ballaigues, Switzerland), was used to shape the root canal to the working length, up to X3 (30/0.07 size/taper) file, following the specifications provided by the manufacturer. During the shaping process, a 30-gauge 2-sided needle (SinaliDent, Shenzhen, Guangdong, China) was kept 2 mm less than the working length utilized for irrigating the solution between each instrumentation file. The samples were grouped into two primary groups (SC and CC), with 32 samples each, based on the irrigation solution, NaOCl/ EDTA (2 mL 3% NaOCl (Modern Medical Equipment LLC, Dubai, United Arab Emirates)) for 1 min after each instrument change, 5 mL 17% EDTA (Cerkamed, Stalwa Wola, Poland) for 1 min and final rinse of 5 mL of distilled water for 1 min) in group SC [6] and NaOCl/ HEDP (2 mL 3% NaOCl/9% Dual Rinse HEDP (Medcem, GmbH, Weinfelden, Switzerland)) for 1 min after each instrument change, 5 mL 3% NaOCl/9% Dual Rinse HEDP for 1 min, and final rinse of 5 mL distilled water for 1 min) in group CC [6]. Immediately prior to instrumentation, 10 mL of the 3% NaOCl were combined with one capsule (0.9 g of etidronate powder) to create the NaOCl/HEDP mixture.

Subsequently, the samples were further divided into four subgroups, each with a total of eight samples, based on the agitation technique used. First, a conventional needle (CN) using a 30-gauge needle, 2-sided vent (SinaliDent, Shenzhen, Guangdong, China) was used throughout all phases of irrigation. The needle was placed 2 mm short of the established working length and moved inside the apical portion of the canal with up-and-down motion. Second, a sonic-driven Endoactivator (EA) (Dentsply Maillefer, Ballaigues, Switzerland) was used to agitate the irrigant using a medium-sized polymer tip (25/0.04) (Dentsply, Maillefer, Ballaigues, Switzerland). It operates at 10,000 revolutions per minute for 60 s, with the tip passively placed two millimeters shorter than the working length into the canal. (UAI) is employed to agitate the irrigant; silver activator tip (Ultra X, Eighteeth, Changzhou Sifary Medical Technology Co., Ltd., Changzhou, China), with a size of 20/0.02 and a length of 21 mm, was attached to an ultrasonic activator (Ultra X, Eighteeth, Changzhou Sifary Medical Technology Co., Ltd., China) at “High Output Power Mode” (frequency 45 kHz). The tip was maintained at 1 mm from the established working length at the center of the canal, and at 2–3 mm, apical–coronal operating motions were executed. Each canal underwent 60 s of ultrasonic agitation. Fourth, the irrigant was agitated with the Er:Cr:YSGG laser using the Er:Cr:YSGG laser (Biolase, Waterlase, Iplus, Irvine, CA, USA), operating at a wavelength of 2780 nm. A 200 µm diameter radial firing tip from Biolase Technology, which was 25 mm long, was used following the instructions of the manufacturer. The absolute calibration factor was 0.85. Panel configuration was set at power = 1.25 W, repetition rate 50 hertz, pulse energy 25 millijoules, and pulse duration 60 microseconds. According to the instructions of the manufacturer, the laser fiber’s tip was positioned 2 mm distant from the apex. A helicoidal motion was performed in contact mode, moving from the apex to the coronal direction at a velocity of 1 mm/s. This movement was conducted in three cycles, with each cycle lasting 20 s. In total, the irradiation time was 60 s. For SC groups, the agitation was done with EDTA irrigant. For all agitation techniques, the irrigation was performed only once before agitation. The volume was utilized, and the sequence of irrigation and agitation is summarized in Table 1.

### 2.3. Root Sectioning and Preparation for SEM and Microhardness Test

The samples were sectioned using diamond discs (22 × 0.4) (Komet Dental., Lemgo, Germany) held under dental loops with a magnification of 3.5×. Longitudinal pre-fracture grooves were created on the buccal and palatal surfaces using a diamond disc with water irrigation. A passive master cone size X3 was inserted in the root canals to prevent contamination during the splitting process. The roots were bisected with a hammer and blade number 11. The samples underwent dryness by vacuum of sputtering coater with gold sputter coating and were examined at 2500× using an Axia Chemisem scanning electron microscope (Thermo Scientific Fisher, Waltham, MA, USA, 2021) at an accelerated voltage of 30 kV. The samples were examined in the coronal, middle, and apical canal sections. Using a permanent marker, horizontal lines were created at the midpoint of each section, which was the region of interest (ROI) for the examined canal wall. This was done to objectively locate each section’s center when inspected under an SEM with 50 µm size of evaluated region.

The images were evaluated by two calibrated examiners based on Hulsmann’s criteria, which assessed the smear layer’s prevalence, amount, and dispersion as follows: score 1 was assigned if no smear layer was present and all dentinal tubules were open, score 2 was assigned if there was a smear layer and more than 50% of the tubules were open, score 3 was assigned if there was smear layer with fewer than 50% of the tubules open, and score 4 was assigned if more than 75% of the tubules were occluded by the smear layer [23].

A Vickers microhardness tester (model: HVS-1000, power: 220 V, 50 HZ, Sr. No. 20221240, Date: 2023.01, Jinan, Shandong, China) was used to measure the surface hardness of the dentin for other halves of each sample, which are horizontally embedded in an autopolymerizing acrylic disc. After removing any surface scratches with a series of fine carbide papers (800, 1200, and 2400 grit), the mounted specimen’s dentin surface was ground flat and smooth. Finally, it was polished using fine grades of composite polishing kit (Shofu Dental, Kyoto, Japan), which used alumina suspension with a size of 50 μm on a rotating felt disc.

**Figure 1:**
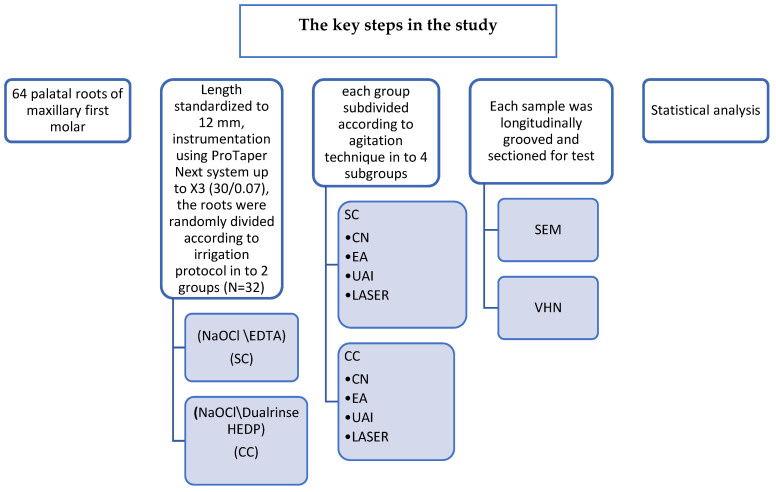
Flow chart summarizing the key steps in the study: SC sequential chelation, CC continuous chelation, CN conventional needle, EA Endoactivator, UAI ultrasonic activated irrigation.

The indentations were created using a Vickers diamond indenter in at least six distinct locations on each specimen. The locations were selected in three regions of the canal wall: apical, middle, and cervical. Measurements were done at each region at 50 and 100 µm depth from the line angle between the lumen of the root canal and the sectioned surface of the canal wall. Indentations were created on the sectioned surface of each sample using a 100 g load for 10 s. The flowchart that summarizes the main steps in the present study is illustrated in Figure 1.

### 2.4. Statistical Analysis

Data were analyzed using SPSS version 26 (SPSS Inc., Chicago, IL, USA). Shapiro–Wilks test was used to assess the normality of data. Kruskal–Wallis, Wilcoxon, and Mann–Whitney U tests were used to compare groups, depths, and sections. The significance level was set at *p* < 0.05. Power calculation of the study sample was based on 95% power with an alpha error of 5% and an effect size of 1.16; eight samples was required for each group [22]. There was a good agreement between the two examiners (weighted kappa = 0.89) for result of smear layer.

## 3. Results

### 3.1. Smear Layer

**Figure 2:**
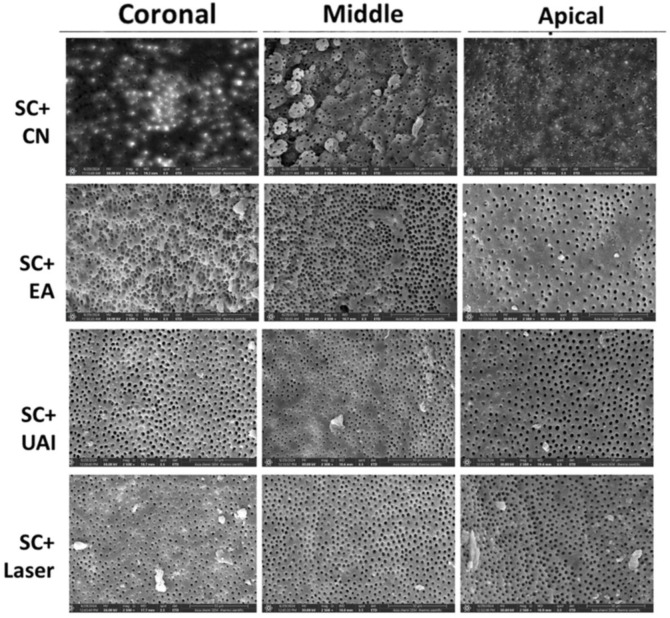
Representative SEM images (magnification 2500×) showing root canal lumen at coronal, middle, and apical sections using SC (sequential chelation) + CN (conventional needle irrigation, EA (EndoActivator), UAI (ultrasonic activated irrigation), and Er:Cr:YSGG laser.

**Figure 3:**
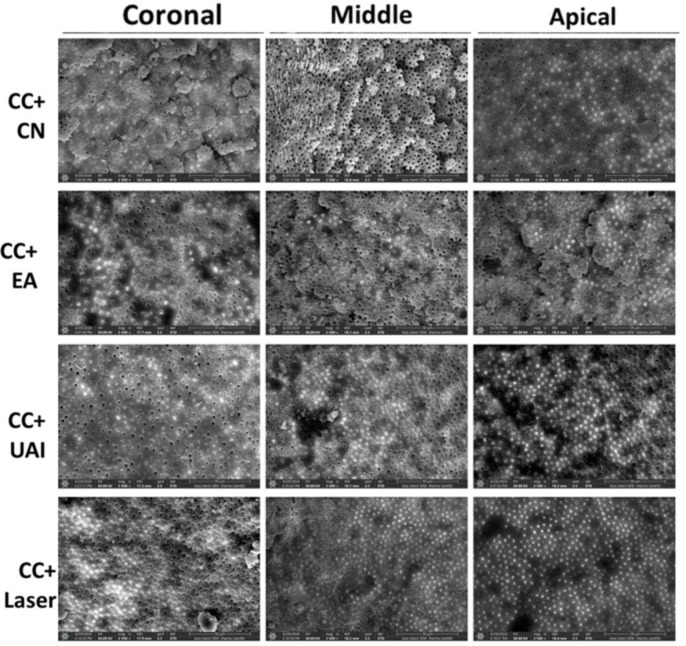
Representative SEM images (magnification 2500×) showing root canal lumen at coronal, middle, and apical sections using CC (continuous chelation) + CN (Conventional needle irrigation, EA (EndoActivator), UAI (Ultrasonic activated irrigation), and Er:Cr:YSGG laser.

There was a good agreement between the two examiners (weighted kappa = 0.89). Table 2 shows the scoring results of the smear layer, including the median and mean rank. Representative scanning electron microscope (SEM) images of all thirds of the SC groups are shown in Figure 2, and the CC groups are shown in Figure 3. A Kruskal–Wallis analysis revealed a statistically significant difference between the different groups (*p* < 0.05).

**Figure 4:**
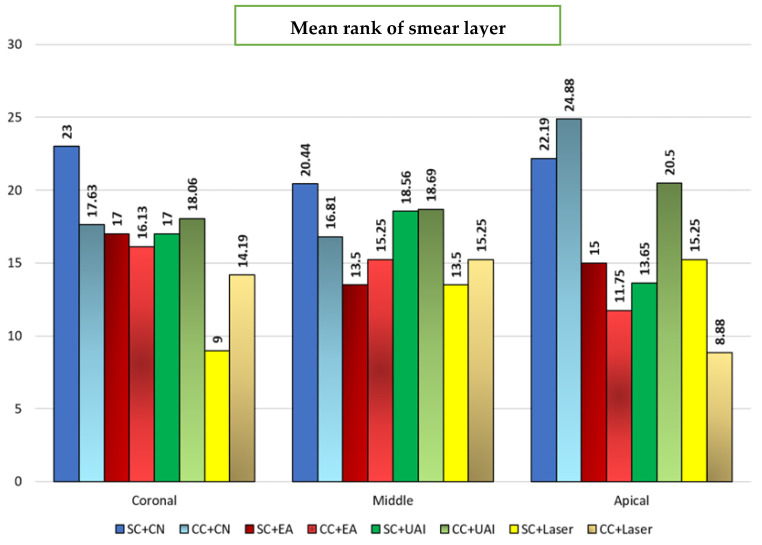
Bar chart illustrating the mean rank of smear layer after SC (sequential chelation) and CC (continuous chelation) in coronal, middle, and apical sections using CN (Conventional needle irrigation), EA (EndoActivator), UAI (Ultrasonic activated irrigation), and Er:Cr:YSGG laser.

The higher scores of smear layers indicated that more smear layers were observed with fewer dentinal tubules opened. For chelators’ comparisons, in the UAI groups, the smear layer scores in the CC were higher than those of SC in the apical third (*p* < 0.05). Also, in laser-agitation groups, there was a considerably higher mean rank of scores in CC compared to SC in the coronal third (*p* < 0.05). Among SC groups, the smear layer scores in the CN group were higher than the laser group in the coronal third (*p* < 0.05). Among CC groups, the smear layer scores in the CN group were higher than those in the EA and laser groups at the apical third. Also, the smear layer scores in UAI were higher than the laser group at the apical third (*p* < 0.05) (Figure 4).

### 3.2. Microhardness

Vickers microhardness values (mean ± S.D.) following the different irrigating procedures are presented in Table 3. For SC groups, there were significant differences in VHN between groups in coronal, middle, and apical sections at both 50 and 100 µm depths (*p* < 0.05). When comparing 50 and 100 µm values of microhardness, there were no significant differences among all subgroups (*p* > 0.05), except in the middle third of laser groups, where there was significantly higher VHN at 50 µm depth compared to 100 µm depth (*p* < 0.05), indicating the dentine is more hard.

For CC groups, there were significant differences in VHN between subgroups in coronal and apical sections at both 50 and 100 µm depths (*p* < 0.05). There were no significant differences in VHN between subgroups in the middle sections at both 50 and 100 µm depths (*p* > 0.05). Comparison of VHN between 50 and 100 µm depths showed no significant differences in all subgroups (*p* > 0.05), except in the apical third of CN and laser groups. There was significantly higher VHN at 50 µm depth compared to 100 µm depth (*p* < 0.05), indicating the dentine is harder.

**Figure 5:**
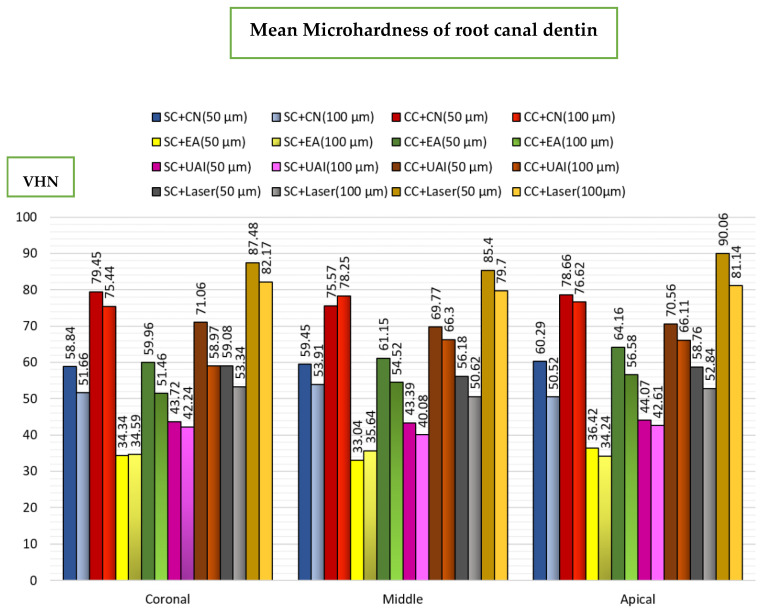
Bar chart illustrating the mean microhardness of root canal dentin after sequential SC and continuous chelation CC in coronal, middle, and apical sections at 50 and 100 µm from the canal surface using: CN (Conventional needle irrigation), EA(EndoActivator), UAI (Ultrasonic activated irrigation), Laser (Er:Cr:YSGG laser).

For chelators’ comparisons, in the CN subgroups, there were significantly higher VHNs of dentine with CC compared to SC in all sections and depths (*p* < 0.05), except at the 50 µm depth in the apical section (*p* > 0.05). Also, there were significantly higher VHNs of dentine with CC compared to SC in all sections and depths (*p* < 0.05), except at the 100 µm depth in the coronal section in both EA and UAI (*p* > 0.05). In laser activation, there were significantly higher VHNs of dentine with CC than SC in all sections and depths (*p* < 0.05) (Figure 5).

## 4. Discussion

The smear layer produced during root canal preparation forms on the canal walls, impeding three-dimensional sealing and efficient cleaning of the root canal system [24]. The study’s results indicated no significant variation in smear layer removal comparing SC versus CC in CN, similar to a prior study that compared SC versus CC without any agitation effect [6], which agrees with our null hypothesis. In contrast, another study showed that HEDP removed the smear layers more effectively than EDTA [25]. This can be attributed to the efficiency of HEDP in reducing the development of the smear layer and decreasing the hard tissue debris that accumulates during the root canal preparation [13]. Conversely, in another study, HEDP was less efficient at a neutral pH than EDTA in eliminating the smear layer; the elevated pH and diminished stability constant of HEDP relative to EDTA resulted in less smear layer clearance [26].

The agitation of irrigation solutions is essential for enhancing the cleanliness of the root canal system. Statistically significant differences in smear layer removal were seen between different agitation methods in the present study (Table 2). The groups of (SC + CN) and (CC + CN) exhibited a substantial smear layer throughout the total canal length compared to other agitation groups, which is similar to a prior study with other irrigants [22]. The present study revealed that all other agitation methods showed much greater efficacy compared to conventional needle applications in eliminating debris and smear layers (Table 2). The major drawback of the conventional needle is that irrigants frequently fail to penetrate the whole root canal. Also, the hydrodynamic shear stresses created by conventional irrigation are not enough to remove the tissue or microbial biofilm that is adhered to the root canal wall [27].

Sonic agitation did not exhibit a significantly different effect compared with ultrasonic agitation, congruent with the other findings, which observed comparable efficacy in removing the smear layer utilizing a #20 ultrasonic tip and a #15 EndoActivator polymer tip, along with a canal featuring a #40/0.02 apical diameter [28], which agrees with our null hypothesis. This finding may be attributed to the restricted capacity of the ultrasonic tip in the root canal, causing agitation of the irrigant solution [29]. In the subgroups of EA and UAI, the variances between coronal and apical thirds may be associated with the problem of restricted apical space for oscillation and agitation of the solutions in the apical third. The findings of this study are consistent with those of another study [30], where removing the smear layer was significantly more effective in the coronal third than in the apical third, disregarding the specific type of agitation technique. Also, there are fewer, smaller-diameter dentinal tubules in the apical third, which were only partially obscured by a thin smear layer [31].

This study’s findings revealed that the EA and UAI enhance irrigation efficiency considerably more effectively than CN, congruent with the other findings, which observed comparable efficacy in removing the smear layer, which showed that XP endo finisher and Ultra-X both demonstrated comparable smear and debris removal, which was more effective than CN [32].

The laser group showed superior efficacy in removing smear layers than other groups on all thirds, with some variations between thirds. The CC group with laser agitation shows the lowest mean rank in the apical third among all others (8.88). This is attributed to the fact that the irrigant receives energy from the laser during agitation, which results in pressure waves and cavitation effects. This results in the irrigant penetrating deeper into the dentinal tubules, which is believed to enhance canal cleaning [33]. The shockwaves generated by dental lasers in root canals can effectively remove the smear layer by promoting the formation of vapor bubbles. These bubbles grow when the laser pulse is initiated and then collapse when the pulse is terminated [34]. During endodontic therapy, a bubble is generated within a restricted geometry, constrained by the walls of the root canal. The expansions and collapses of the bubble generate fluid streaming, which may explain the cleaning efficacy of laser-induced irrigation [35]. According to the experiment’s findings, the Er,Cr,YSGG laser had the best smear layer cleaning performance across the entire root canal region. The irrigation solution can fully flow in the root canal system to accomplish the disinfection and smear layer removal effect because the high-energy water molecules damage the target cells and penetrate the “airlock effect” region of the root apical segment [36]. It has been noted that the inorganic dentin components can be melted by applying an Er,Cr,YSGG laser to the root canal, which effectively removes debris from the root canal wall [37].

The microhardness test is used to measure the surface hardness of the dentin. Significant differences in VHN between groups in coronal, middle, and apical sections at 50 and 100 µm depths (*p* < 0.05) were observed between groups, which is in disagreement with the null hypothesis. Tested specimens treated with HEDP exhibited markedly greater microhardness values than those treated with EDTA, corroborating a previous study [9]. Regardless of the agitation technique used, there was significantly higher VHN after CC compared to the SC irrigation protocol. This reference to CC retains greater mechanical integrity of dentin than SC, probably because it removes less calcium from the dentin. An investigation was conducted to assess the effects of chelating agents on calcium depletion and their subsequent effects on the microhardness of dentin. The results indicated that an increase in calcium loss from root dentin led to a decrease in the microhardness of root dentin [38].

In this study, the application of EDTA resulted in a significant decrease in dentin surface microhardness compared to HEDP. This reduction can be attributed to the considerable loss of mineral content and hydroxyapatite in the intertubular dentin, which adversely affects the hardness of the human dentin structure [8], while partial degradation of the surface collagen fibers is exposed by HEDP and proteolyzed by sodium hypochlorite [39].

Regarding the impact of the agitation method on the demineralization efficacy of each chelating irrigant, the root dentin exposed to CN and laser agitation showed greater hardness compared to EA and UAI, with the highest hardness recorded in the roots of the laser-agitation groups. The agitating irrigants with Er:Yag laser (2-watt power, 15_ hertz frequency) led to a decrease in micro-hardness compared to agitation without the laser, which agrees with our findings [40]. Also, another study revealed that the agitation with PIPS did not result in any further reduction in dentin microhardness compared to non-agitated samples [41]. This implies that the irrigation solution, not the agitation technique, changed dentin mineral content and microhardness. The degree of these alterations depends upon the composition of the laser energy and the tissue’s density and absorption properties [42]. According to our findings, irrigation solutions activated with sonic and ultrasonic agitation decreased microhardness because ultrasonic agitation improves the ability of irrigant solutions to penetrate the dentinal tubules [43].

This study is constrained by the in vitro environmental conditions. Thus, the acquired findings cannot be readily applied to clinical situations. The tests were conducted at room temperature rather than at body temperature. Moreover, it is important to remember that SEM images are obtained from a specific area of the canals and may not be representative of the whole root canal wall surface. A further limitation of SEM technology is that it yields a two-dimensional image, which precludes the assessment of the thickness of the smear layer and debris. Ideally, a longitudinal observation of the canal using micro-CT is regarded as a fundamental requirement to study the smear layer removal procedures. Other possible limitations are variations in parameters such as the range of stresses applied for microhardness testing, individual variability in the dentin characteristics among specimens, method of delivering the irrigating solution, and differences in measuring procedures, which might lead to discrepancies in the results. Future in vivo studies are necessary to investigate the significance in clinical practice of using these irrigation procedures on the elimination of smear layers and the ultrastructure of dentin.

## 5. Conclusions

The results indicate that CC combined with laser agitation is the most effective technique for smear layer removal. CC was comparable to SC in removing the smear layer from the root canal dentin wall. Nevertheless, neither solutions nor agitation methods could fully eradicate the smear layer from root canals. CC had a significantly lower detrimental effect on dentin microhardness, mainly with CN and laser agitation, compared to the more traditional SC. In this study, CC resulted in significantly higher dentin microhardness than SC, which may encourage CC in clinical practice and improve the outcome of endodontic treatment.

## Figures and Tables

**Table 1:** The protocols of irrigation and agitation used in each group of the study.

Group	The Sequence of Irrigation and Agitation Protocol
SC	1. 2 mL 3% NaOCl for 1 min after each instrument change. 2. SC + CN subgroup: 5 mL 17% EDTA for 1 min. SC + EA, SC + UAI and SC + Laser subgroups: 5 mL 17% EDTA for 1 min with 3 cycles of agitation for 20 s each. 3. Final rinse of 5 mL distilled water for 1 min.
CC	1. 2 mL 3% NaOCl/9% Dual Rinse HEDP for 1 min after each instrument change. 2. CC + CN subgroup: 5 mL 3% NaOCl/9% Dual Rinse HEDP for 1 min. CC + EA, CC + UAI and CC + Laser subgroups: 5 mL 3% NaOCl/9% Dual Rinse HEDP for 1 min with 3 cycles of agitation for 20 s each. 3. Final rinse of 5 mL distilled water for 1 min.

SC = sequential chelation, CC = continuous chelation, CN = conventional needle, EA = Endoactivator, UAI = ultrasonic activated irrigation.

**Table 2:** Median and mean rank of smear layer scoring in the coronal, middle, and apical thirds of the root canal for all groups.

Group	Coronal		Middle		Apical	
	Median	Mean Rank	Median	Mean Rank	Median	Mean Rank
SC + CN	2.00	23.00 ^a,^*	2.00	20.44	2.50	22.19
SC + EA	1.50	17.00	1.50	13.50	2.00	15.00
SC + UAI	1.50	17.00	2.00	18.56	2.00	13.56
SC + Laser	1.00	9.00 ^a,^*	1.50	13.50	2.00	15.25
CC + CN	2.00	17.63	2.00	16.81	3.00	24.88 ^a,b,^*
CC + EA	2.00	16.13	2.00	15.25	2.00	11.75 ^a,^*
CC + UAI	2.00	18.06	2.00	18.69	3.00	20.50 ^c,^*
CC + Laser	2.00	14.19	2.00	15.25	2.00	8.88 ^b,c,^*

* Kruskal–Wallis test. Identical superscript lowercase letters indicate significant differences among relevant groups in each chelator (Mann–Whitney U test for pairwise comparison). Identical superscript lowercase letters indicate significant differences among relevant groups in between chelators (Mann–Whitney U test for pairwise comparison (*p* < 0.05)). SC sequential chelation, CC continuous chelation, CN conventional needle, EA Endoactivator, UAI ultrasonic activated irrigation.

**Table 3:** Average Vickers microhardness values (mean ± standard deviation) of dentin after different irrigating protocols.

Section and Depth	Group	Mean (VHN)	SD	Group	Mean (VHN)	SD
Sequential Chelation				Continuous Chelation		
Coronal_50	SC + CN	58.84 ^a,^*	18.69	CC + CN	79.45	6.87
	SC + EA	34.34 ^a,b,^*	6.49	CC + EA	59.96 ^a,^*	18.85
	SC + UAI	43.72	8.99	CC + UAI	71.06 ^b,^*	25.56
	SC + Laser	59.08 ^b,^*	7.61	CC + Laser	87.48 ^a,b,^*	14.81
Middle_50	SC + CN	59.45 ^a,^*	14.93	CC + CN	75.57	6.84
	SC + EA	33.04 ^a,b,^*	9.87	CC + EA	61.15	22.87
	SC + UAI	43.39	6.71	CC + UAI	69.77	20.67
	SC + Laser	56.18 ^b,^*	6.26	CC + Laser	85.40	8.62
Apical_50	SC + CN	60.29 ^a,^*	16.79	CC + CN	78.66	0.51
	SC + EA	36.42 ^a,b,^*	6.46	CC + EA	64.16 ^a,^*	13.10
	SC + UAI	44.07	1.94	CC + UAI	70.56 ^b,^*	23.00
	SC + Laser	58.76 ^b,^*	10.36	CC + Laser	90.06 ^a,b,^*	20.64
Coronal_100	SC + CN	51.66 ^a,^*	14.81	CC + CN	75.44	3.08
	SC + EA	34.59 ^a,b,^*	8.95	CC + EA	51.46 ^a,^*	22.49
	SC + UAI	42.24	9.74	CC + UAI	58.97 ^b,^*	24.97
	SC + Laser	53.34 ^b,^*	7.23	CC + Laser	82.17 ^a,b,^*	12.24
Middle_100	SC + CN	53.91 ^a,^*	13.09	CC + CN	78.25	5.84
	SC + EA	35.64 ^a,b,^*	4.76	CC + EA	54.52	25.22
	SC + UAI	40.08	11.24	CC + UAI	66.30	19.32
	SC + Laser	50.62 ^b,^*	5.18	CC + Laser	79.70	10.34
Apical_100	SC + CN	50.52 ^a,^*	11.10	CC + CN	76.62 ^a,^*	1.41
	SC + EA	34.24 ^a,b,^*	10.99	CC + EA	56.58 ^a,b,^*	15.60
	SC + UAI	42.61	7.52	CC + UAI	66.11	21.94
	SC + Laser	52.84 ^b,^*	5.05	CC + Laser	81.14 ^b,^*	22.42

* Kruskal–Wallis test. Identical superscript lowercase letters represent significant differences among the relevant groups in each section and depth (Mann–Whitney U test (*p* < 0.05). SC sequential chelation, CC continuous chelation, CN conventional needle, EA Endoactivator, UAI ultrasonic activated irrigation.

## Data Availability

The original contributions presented in this study are included in the article. Further inquiries can be directed to the corresponding author.
